# Otolaryngology and Speech Therapy evaluation in the assessment of oropharyngeal dysphagia: a combined protocol proposal

**DOI:** 10.1590/S1808-86942011000200010

**Published:** 2015-10-19

**Authors:** Patrícia Paula Santoro, Cristina Lemos Barbosa Furia, Ana Paola Forte, Elza Maria Lemos, Roberta Ismael Garcia, Raquel Aguiar Tavares, Rui Imamura

**Affiliations:** 1 PhD in Medicine - FMUSP (University of São Paulo Medical School), ENT at the FMUSP University Hospital. Head of the Dysphagia Ward - ENT Dept. FMUSP; 2 PhD in Sciences - Oncology - FMUSP, Supervisor of the Dysphagia Specialization Program FMUSP - Dept. of Speech Therapy, Physical Therapy and Occupational Therapy - FMUSP. Coordinator of the Dysphagic Patient Care Program - São Paulo City Hall Health Secretariat - Southeast region; 3 MSc in Sciences - FMUSP, Speech Therapist - Dysphagia Ward - ENT Dept. - FMUSP; 4 PhD student in Medicine - FMUSP, Physician at the Dysphagia Ward - ENT Detp. FMUSP; 5 PhD student in Medicine - FMUSP, Physician at the Dysphagia Ward - ENT Detp. FMUSP; 6 PhD in Medicine - FMUSP, Physician at the Dysphagia Ward - ENT Detp. FMUSP; 7 PhD in Medicine - FMUSP, Assistant Physician - ENT Dept. FMUSP. Head of the Oro-pharyngology Group - FMUSP

**Keywords:** evaluation, deglutition disorders, laryngoscopy

## Abstract

Dysphagia is a symptom associated with an array of anatomical and functional changes which must be assessed by a multidisciplinary team to guarantee optimal evaluation and treatment, preventing potential complications.

**Aim:**

The aim of the present study is to present the combined protocol of clinical and swallowing videoendoscopy carried by ENT doctors and speech therapists in the Dysphagia Group of the ENT Department - University Hospital.

**Materials and Methods:**

Retrospective study concerning the use of a protocol made up of patient interview and clinical examination, followed by an objective evaluation with swallowing videoendoscopy. The exam was performed in 1,332 patients from May 2001 to December 2008. There were 726 (54.50%) males and 606 (45.50%) females, between 22 days and 99 years old.

**Results:**

We found: 427 (32.08%) cases of normal swallowing, 273 (20.48%) mild dysphagia, 224 (16.81%) moderate dysphagia, 373 (27.99%) severe dysphagia and 35 (2.64%) inconclusive exams.

**Conclusion:**

The combined protocol (Otolaryngology and Speech Therapy), is a good way to approach the dysphagic patient, helping to achieve early and safe deglutition diagnosis as far as disorder severity and treatment are concerned.

## INTRODUCTION

Dysphagia happens because of disorders in any of the phases of swallowing (preparatory, oral, pharyngeal and/or esophageal). Swallowing unbalance may cause severe pulmonary complications, malnutrition and dehydration - all associated with high morbidity and mortality rates^1^.

Numerous studies have been carried out aiming at establishing preventive measures in order to mitigate these complications. Thus, it is very important to very carefully assess swallowing, with clinical and complementary exams involving objective tests such as the Videoendoscopic Swallowing Study (VESS) 2, 3. When these assessments are carried out by an integrated multidisciplinary team, diagnosis is more precise and the patient benefits the most.

The guided interview for swallowing disorders aims at shedding some light on the etiological and clinical aspects of the disorder, as well as on patient performance during feeding. Thus, the acquisition of this data enables the examiner to raise hypotheses in order to obtain a possible etiological diagnosis, have knowledge about the presence of associated disorders, cognitive aspects integrity, bronchipulmonary disorders and the patient's general clinical status.

The clinical exam aims at helping us understand swallowing dynamics, and it is made up of specific assessment procedures of the anatomical structures involved and the functioning of its phases^4^. We start by assessing posture, muscle tone, mobility and sensitivity of the structures which are part of the swallowing process, and this is considered an indirect assessment, because there is no food involved. Afterwards, we assess it with food in different quantities and consistencies, which aims at analyzing the dynamics of swallowing, considering its different phases^5^.

Clinical evaluation should be complemented by objective methods. In our clinic we routinely do the VESS, which enables the detection of possible anatomical and/ or functional changes of the structures involved in swallowing. It also enables the examiner to assess swallowing efficacy and the very integrity of the mechanisms which protect the airway, simulating a meal with food of different consistencies and quantities, keeping a direct view through the fiberoptic device^3^, ^6^, ^7^. It is a good test to track deglutition, involving a technology that is simple, inexpensive and practical. Lately, VESS has become a validated technique to assess the pharyngeal phase of deglutition, proving to be as sensitive and specific as the traditional video-deglutogram, in many of its versions^8^.

Studies have shown that the speech evaluation is sensitive to detect and classify changes to the preparatory, oral and pharyngeal phases. These data, added to the VESS analysis enables the physician to do an objective evaluation of the pharyngeal phase of deglutition and, consequently, a more complete and more accurate diagnosis^9^.

The goal of the present paper is to introduce the clinical and VESS assessment protocol created in a joint work between otolaryngologists and speech therapists at the Dysphagia Ward of the ENT Department of our Institution.

## MATERIALS AND METHODS

The present study protocol was approved under record # 412/02 by the Ethics Committee for Research Project Analysis - CAPPesq, of our Institution. All participants in the study protocol were previously instructed by the researcher in charge, in an accessible language, respecting all guidelines established by the aforementioned committee, and they all freely accepted to participate in the study.

From May of 2001 through December of 2008 we carried out 1,332 evaluations using the Joint Protocol for Deglutition Assessment.

The protocol was made up by the patient's identification, interview, integrated speech and ENT tests and objective evaluation using the VESS.

### Interview

In the initial interview we obtained information about: baseline disorders, main complaints, history and evolution of the clinical manifestations, tests and treatments done, general health condition, specific deglutition-related complaints (oral, pharyngeal and esophageal phases), feeding conditions (consistency, posture, utensils and complications), feeding path (oral or alternative), vocal characteristics and issues associated with the nutritional status and repetition pneumonias.

### Clinical Evaluation - Subjective and Objective

In both assessments which were carried out (subjective and objective), the procedure used to prepare the consistencies, patient posture and diet used to assess deglutition were all similar. The sole difference between the procedures was the use of a dye (blue edible dye) for the videoendoscopic evaluation.

### Food consistencies

We used foodstuff dyed with blue dye (edible dye) in the following consistencies: liquid, thick liquid, paste and solid). The liquid consistency was that of filtered water at room temperature. In order to thicken the liquid and make the paste, we used starch-based food thickener, added to filtered water at room temperature, respecting the standardization of the *Thick-easy* (Fresenius-Kabi) product: thick liquid: 4.5g of the thickener with 100mL of water; Thick: 9.0g of the thickener with 100mL of water. The solid consistency was obtained by giving the patient the "salt and water" snack (Figs. 1A and 1B).

**Figure 1a:**
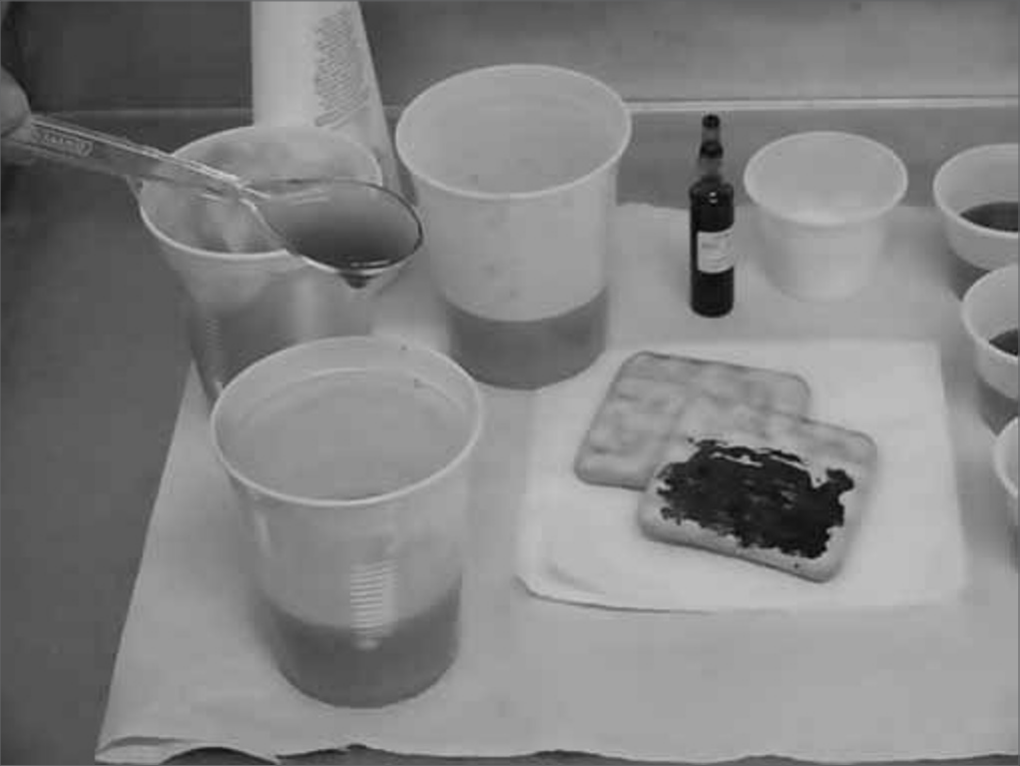
Thick liquid preparation using a food thickener.

**Figure 1b:**
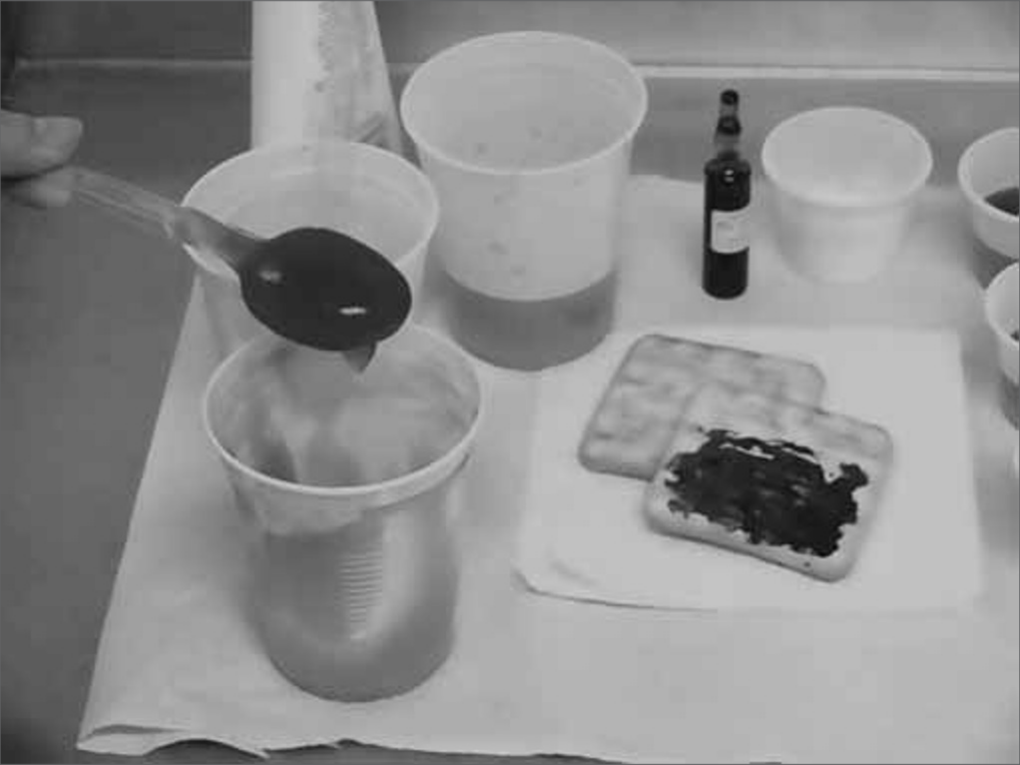
Pasty consistency preparation using a food thickener.

### Patient position

**Figure 2:**
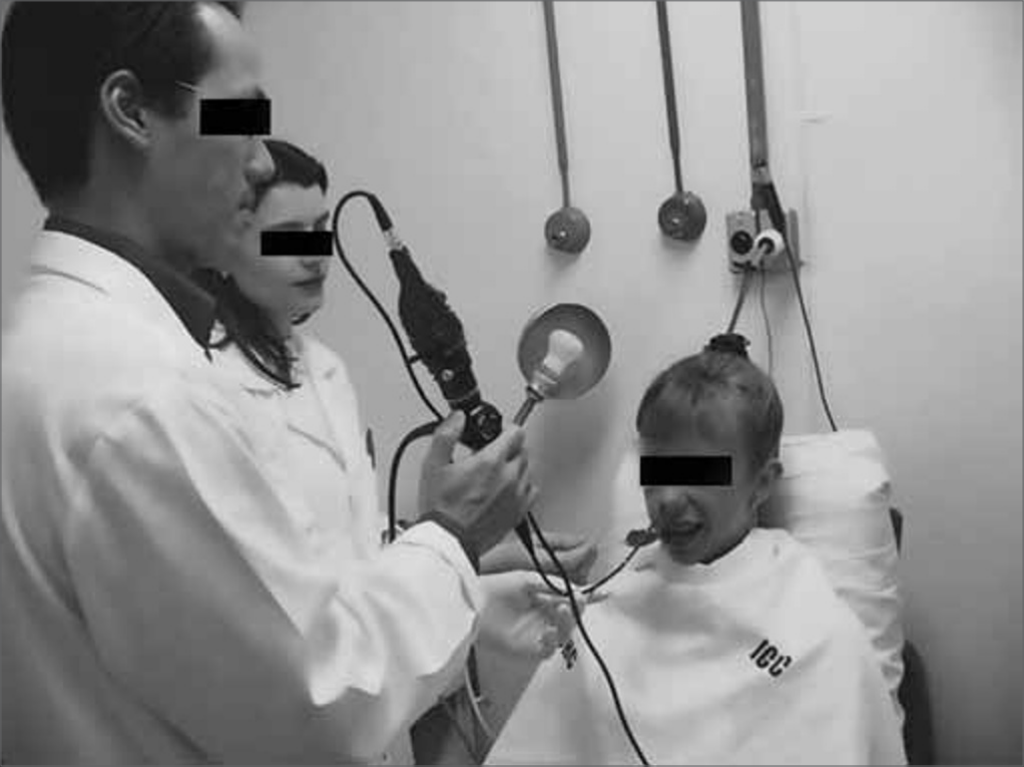
Positioning the patient in order to do the deglutitions videoendoscopy. Patient seating down with the neck in mild ventroflexion.

In order to do the assessments, the patient was instructed to remain seated, keeping his head slightly down - simulating the eating position (Figure 2). As for patients in bed, the test was done with the bed tilted upwards as close as possible to 90°.

### Foodstuff

The food was given to the individuals according to the following sequence:

• Liquids (3mL, 5mL, 10mL and free gulps);
• Thickened liquid stuff (3mL, 5mL, 10mL and free gulps);
• Paste (3mL, 5mL and 10mL);
• Solids (¼ of a “salt and water” snack; corresponding to 3.6cm^3^ of solid).

Liquids, thickened liquids and pasty foodstuff were given to the individuals in a metered syringe, and the sample was placed directly in the patient's mouth. The solid food was given in a standard size. At the end of the assessment, when possible, we offered the patients some liquid and thickened liquids.

The consistency sequence and the amount of food given varied according to the data we obtained from the interview and during the clinical evaluation, so as to avoid the risk of aspiration.

### Subjective Clinical Assessment

The deglutition clinical assessment was done by the speech therapist, and made up of two distinctive phases: indirect assessment (without giving food) and direct assessment (with food given).

On the first stage, the exam was made by means of objective questions, observation, touch and asking the patient to move in order to check for the following aspects:

• General health status (motor, cognitive and communication);
• Breathing: type and mode
• Phono-articulatory organs (tone, posture and mobility of tongue, lips, cheeks, mandible and soft palate; teeth; touch, thermal, and taste sensitiveness of the facial, intraoral and pharyngeal regions; hard palate; vocal quality);
• oral (vomit and cough);
• Saliva deglutition (automatic, voluntary, sialorrhea; xerostomia, gagging, cough, voice quality change - “wet voice”).

The clinical evaluation with the diet was done giving the patient food in the quantities and consistencies discussed above. By means of observation and touch, we assessed the following aspects: mouth opening spontaneity, lip closure capacity; chewing efficiency; tongue mobility efficiency in preparing the food cake; deglutition reflex triggering at the onset of the pharyngeal phase; presence and efficiency of the laryngeal elevation; hawking; gagging or cough before, during or after swallowing; presence of food residues in the oral cavity.

### Objective Clinical Assessment (VESS)

The otolaryngologist assessed the patient's structural Pharyngo-laryngeal anatomy and the deglutition function using the fiberoptic device - VESS. The test was carried out with the help of a speech therapist.

All the exams were recorded in a DVD, which enabled the examiners to review and digitalize the images obtained. We used conventional video-endoscopic equipment made up of:

• SEMP^®^, 10" color TV Set model 1022FAVU11;
• Panasonic^®^ DMR - E55 DVD video recorder
• Micro camera Toshiba^®^ A43 micro camera coupled to a Machida^®^ CA - 34VS2 endoscope coupler;
• Machida^®^ 3,2mm flexible fiberoptic scope
• Welch Allyn^®^ Metal Hilide power source

The fiberoptic device was introduced through the patient's broader nasal cavity, without the use of topical anesthesia, so as not to interfere in Pharyngo-laryngeal sensitivity.

The VESS exam routine followed the protocol described by Langmore^10^:

We initially assessed the rhinopharynx, a panoramic view of the Pharyngo-laryngeal area and assessment of its sensitivity. We observed: velopharyngeal closure upon phonation and deglutition; saliva and secretion clearance, saliva aspiration signs, glottic closure and vocal fold mobility. Thus, the fiberoptic scope was broadly moved in order to enable a detailed structural assessment, similarly to what is routinely done by otolaryngologists (Figs. 3A, 3B and 4A, 4B).

**Figure 3a:**
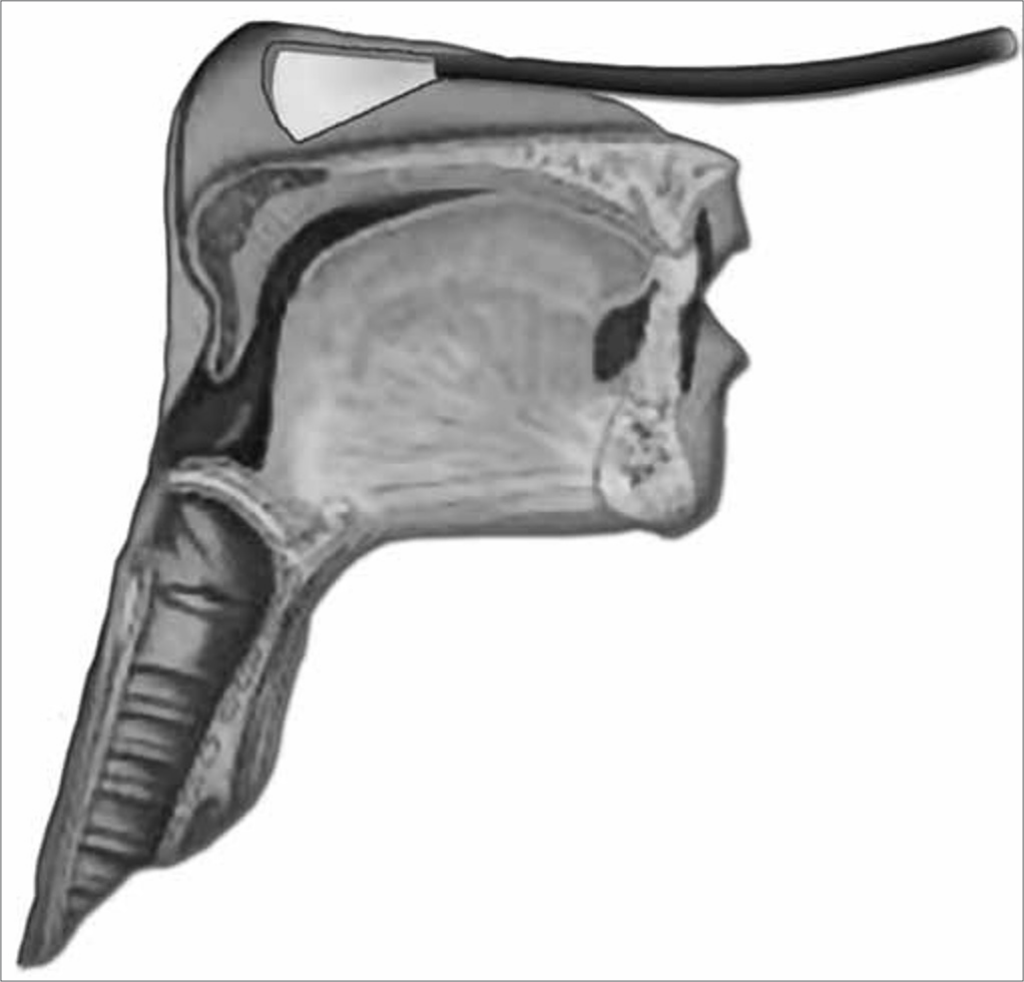
Positioning the fiberoptic device in order to assess the rhinopharynx structure.

**Figure 3b:**
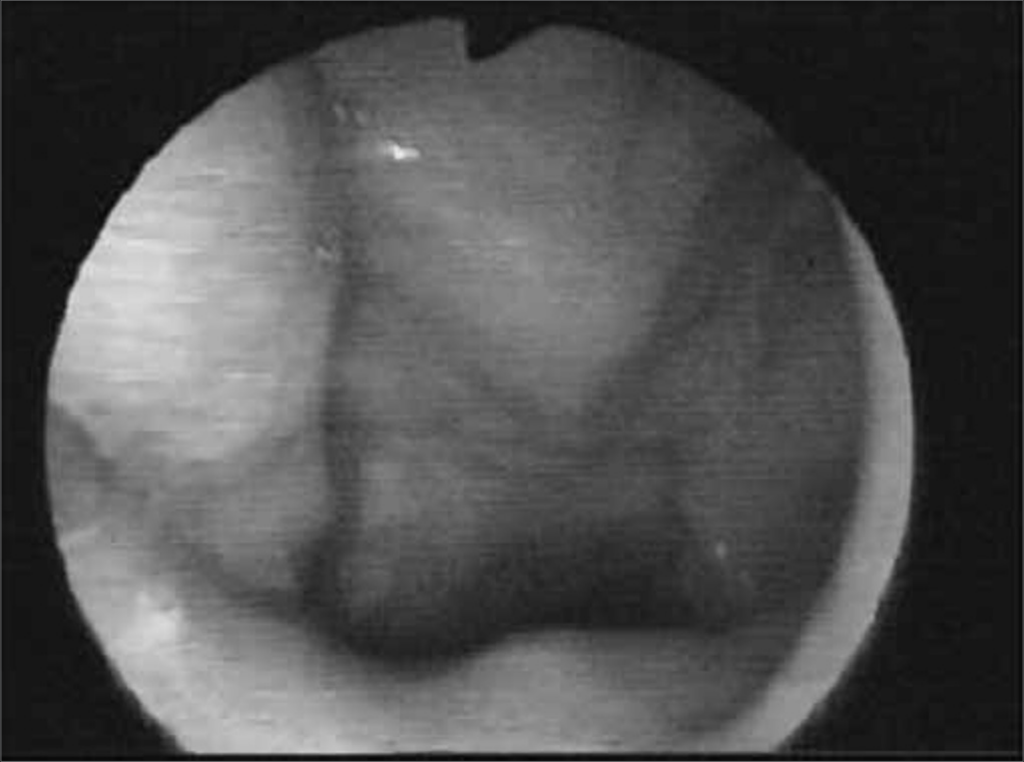
Rhinopharynx structures and velopharyngeal closure.

**Figure 4a:**
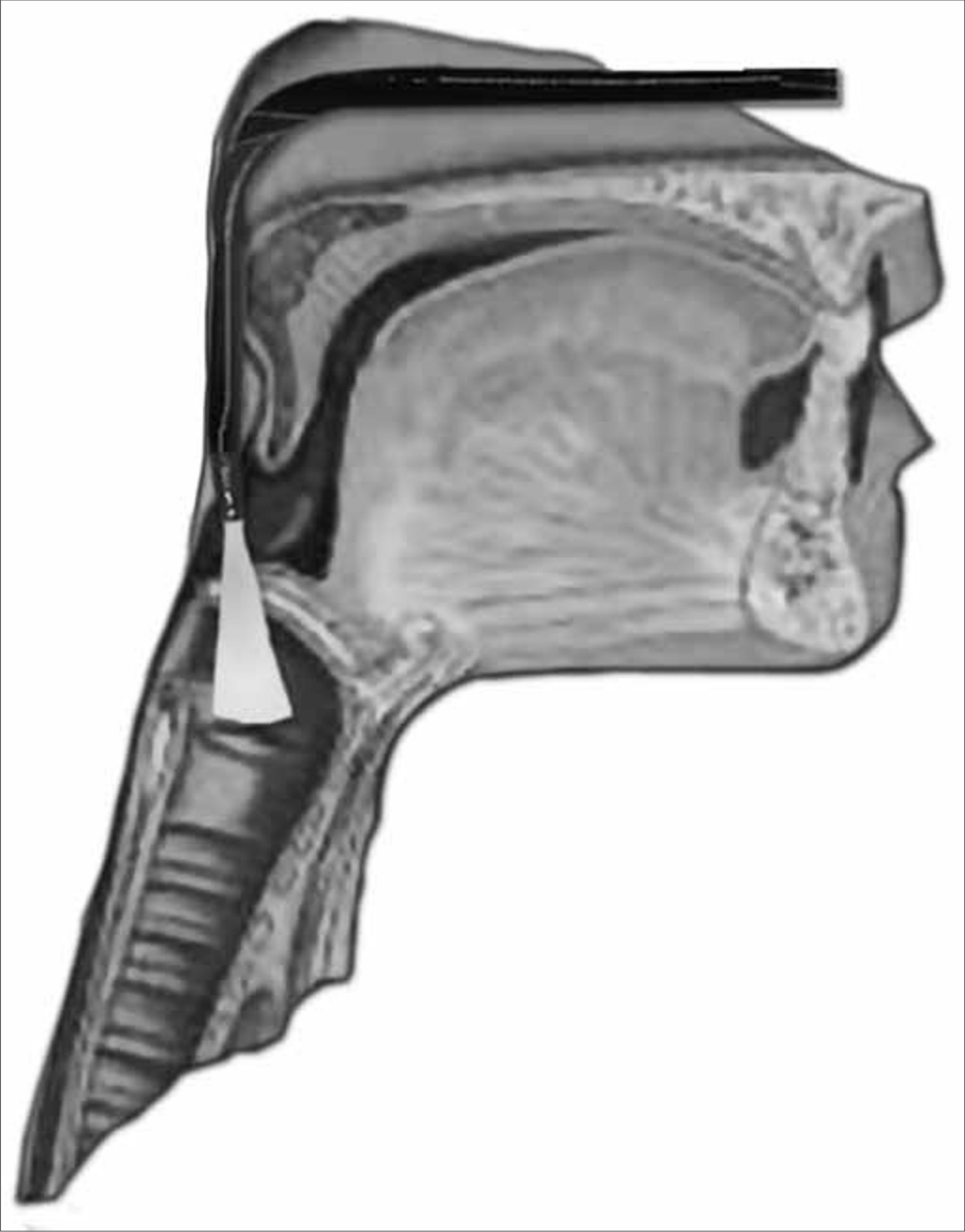
Positioning the fiberoptic device in order to carefully examine the pharynx and larynx.

**Figure 4b:**
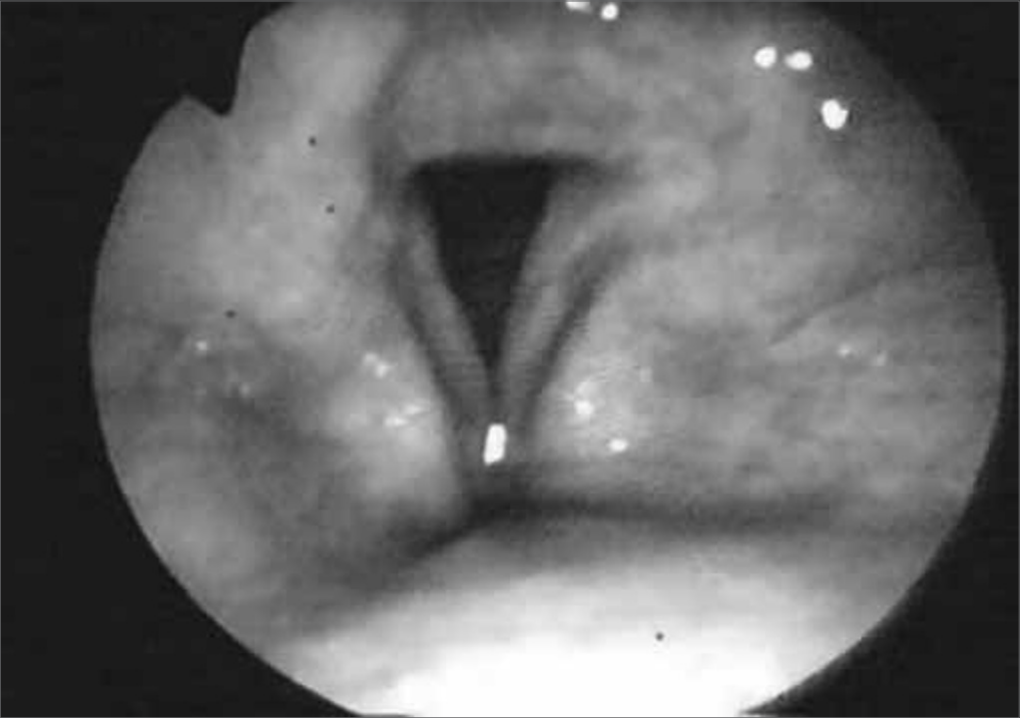
Pharyngolaryngeal structures and glottic closure.

Afterwards, we checked for swallowing capacity and limitations concerning the four types of food aforementioned. This phase also involved a broad panoramic view of the pharynx, larynx and subglottic region at the end of the assessment, looking for aspiration before, during or after swallowing. We assessed issues associated with the main events of the oral and pharyngeal phases of deglutition: tongue base mobility; food containment in the oral cavity; nasal food reflux; presence of food residues after deglutition and where it happened; laryngeal penetration; laryngotracheal aspiration of the food cake and the number of deglutitions needed for the complete clearance of the food cake. We also assessed posture and upper airway protection maneuvers (chin down; head back; turned or tilted head; and also supraglottic, super-supraglottic maneuvers, stress, Mendelson and multiple deglutitions) tested during the assessment of deglutition for each specific case and its efficacy.

For the function assessment of deglutition, the fiberoptic scope was placed upwards in the pharynx, behind the uvula. Considering that at the exact time of deglutition, the larynx moves up and anteriorly, and because of that we tried to avoid touching pharyngolaryngeal structures which could trigger the gag reflex and compromise the deglutition dynamics (Figs. 5A and 5B).

**Figure 5a:**
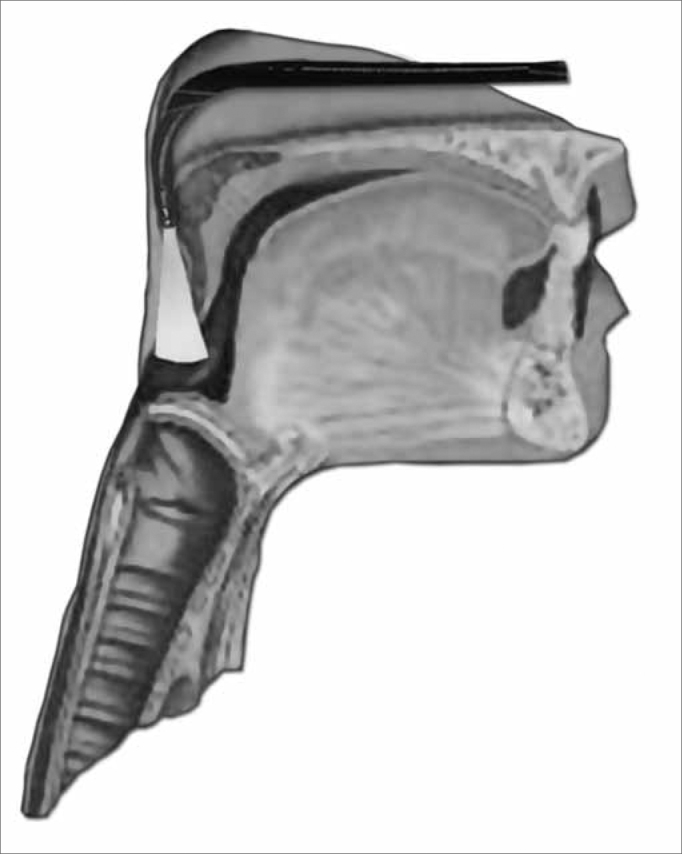
Positioning the device in order to assess the deglutition function.

**Figure 5b:**
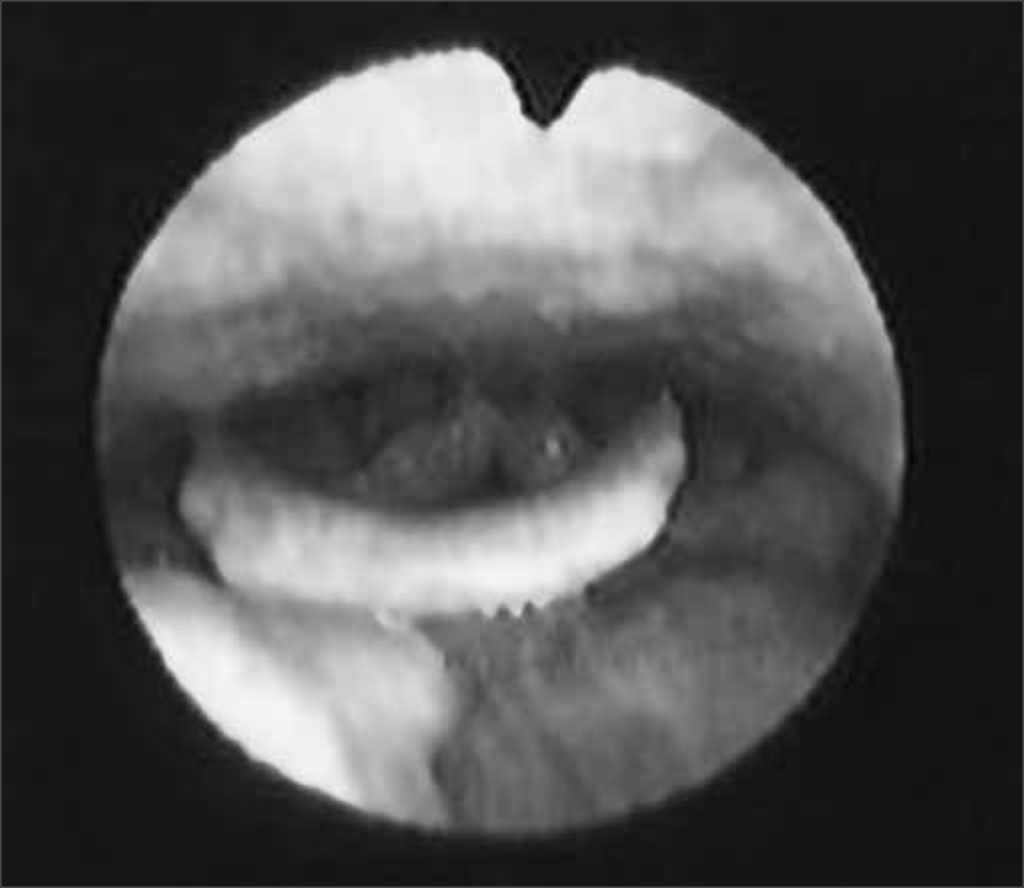
Panoramic view of the Pharyngo-laryngeal structures for a functional assessment of deglutition.

As the pharyngeal walls contracted over the fiberoptic scope, light was blocked and reflected, consequently preventing us from having a direct view of the deglutition events, so called *white-out* phase (Figs. 6A and 6B).

**Figure 6a:**
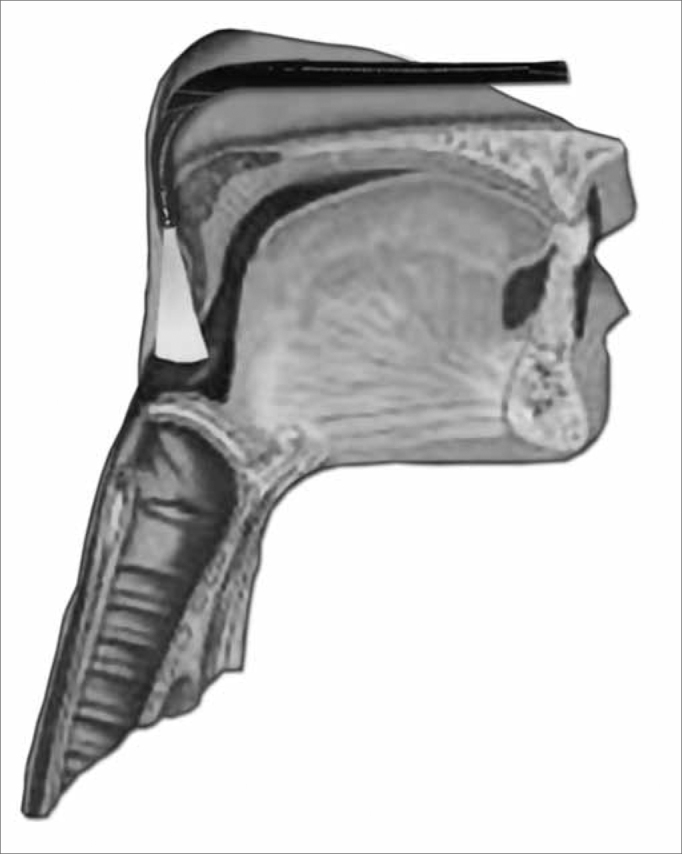
White out phase: light blockage because of pharyngeal contraction.

**Figure 6b:**
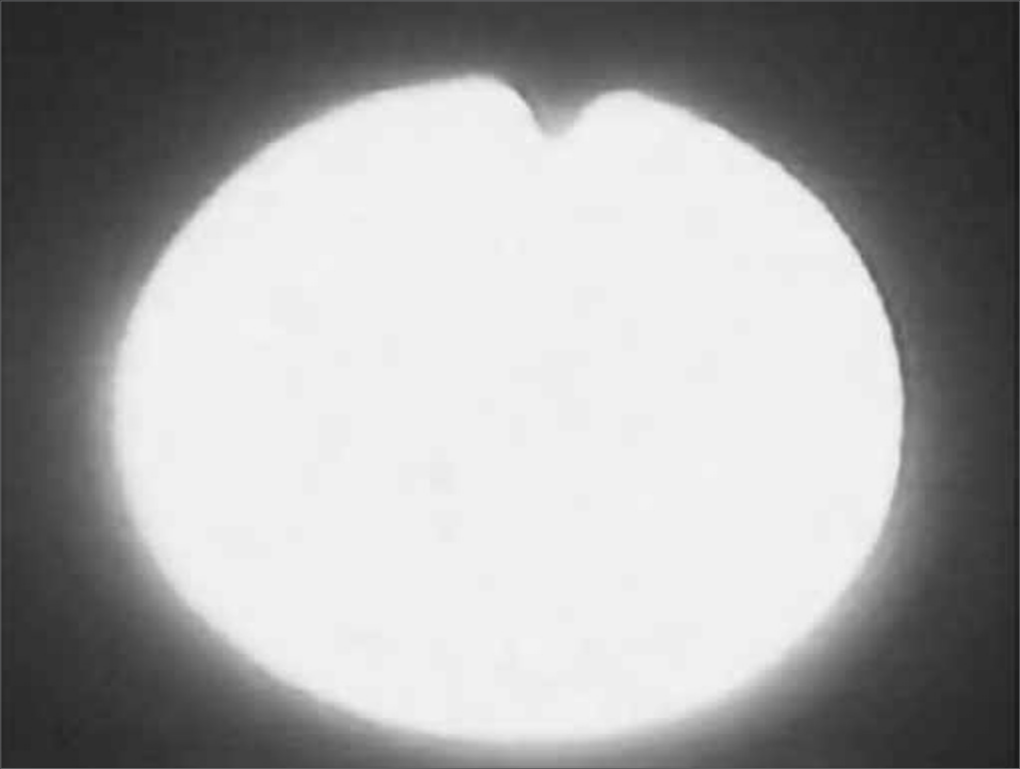
Endoscopic view of the White out phase.

And finally, the observations done during the two VESS stages enabled the endoscopic classification of the oropharyngeal dysphagia.

### Qualitative parameters observed

*VESS structural assessment (without food being given):* velopharyngeal closure; saliva stasis in valleculae and pyriform recesses, signs of saliva aspiration, Pharyngolaryngeal sensitivity reduction; glottic closure changes and/or vocal fold movements, pharyngeal and laryngeal signs suggesting lesion caused by gastroesophageal reflux.

#### Functional assessment (with food) by VESS

Deglutition oral phase: changes to base tongue mobility and early food cake escape.

Deglutition pharyngeal phase: nasal reflux, post-deglutition residue, laryngeal penetration, laryngotracheal aspiration, cough reflex.

Upper airway protection and posture maneuvers test.

### Observed quantitative parameters

*Number of deglutitions:* spontaneous, requested and the total number of deglutitions for the complete clearance of the food cake.

Clinical-endoscopic classification of dysphagia

Clinical-endoscopic classification of dysphagia^11^: we did it considering all the variables obtained on the previous stages of the test:

• Normal deglutition (level 0): normal oral contention, reflexes present, no salivary stasis, feeding and aspiration, fewer than three attempts to push for food clearance.
• Mild dysphagia (level 1): small post-deglutition stasis, less than three attempts to clear the food cake, no nasal regurgitation and laryngeal penetration;
• Moderate dysphagia (level 2): moderate salivary stasis, more post-deglutition stasis, more than three attempts to push for food cake clearance; nasal regurgitation; reduction on laryngeal sensitivity with penetration in the laryngeal vestibule; however without laryngotracheal aspiration;
• Severe dysphagia (level 3): major salivary stasis; important worsening in post-swallowing residues, bad or absent propulsion, nasal regurgitation, tracheal aspiration.

## RESULTS

From May 2001 through December of 2008, we did 1,332 evaluations by means of the Joint Protocol for Deglutition Assessment, involving 726 (54.50%) males and 606 (45.50%) females. Their ages varied between 22 days and 99 years, with a mean age of 59.4 years. We found 427 (32.08%) patients with normal deglutitions, 273 (20.48%) with mild dysphagia, 224 (16.81%) with moderate dysphagia, and 373 (27.99%) patients with severe dysphagia. In 35 (2.64%) patients it was not possible and/or conclusive to do the VESS, and the main reasons which made the exam impossible were: not seeing the glottis (Tumor, laryngomalacia...), hyperreflexia / nausea / vomits, vasovagal reflex, tachi-dyspnea, sleepiness (medication side effect or cognitive state oscillation), refusal to eat, intense crying, unwillingness to collaborate.

## DISCUSSION

Dysphagia is a symptom which involves a number of anatomical and functional changes, which should be approached in a multidisciplinary fashion. Many health-care specialists must work together in order to guarantee proper assessment and access to all the factors associated with it, as well as take all the necessary measures which help control the dysfunction, preventing the potential complications such as malnutrition, dehydration and aspiration pneumonia.

Talking specifically about oropharyngeal dysphagia, the multidisciplinary team must^12^:

Make sure dysphagia is present,

Identify possible etiologies for the dysfunction,

Rule out structural components (cysts, tumors, vocal fold paralysis, and others),

Make sure the anatomical and functional structures involved in the oropharyngeal deglutitions are intact,

Assess the risk of aspiration pneumonia.

In this context, it is necessary to discuss the importance of the joint work between otolaryngologists and speech therapists, trying to clarify data concerning anamnesis and physical exam, paying attention to the comorbidities and changes to anatomical structures and cranial nerves involved in the deglutition process. To carry out a specific clinical assessment by means of observing the patient, investigating the functionality of the phono-articulatory organs and following on the feeding time of these patients, when possible, assessing posture, handling of utensils, difficulties concerning consistencies and quantities, as well as identifying deglutition facilitating maneuvers^13^; to add to the assessment with data from exams, such as the VESS, doing an objective assessment of the associated functional and anatomical structures; to propose clinical and/or surgical approaches which aim at helping deglutition; to lead the rehabilitation process of the dysphagic patient, with training exercises, protective maneuvers and maneuvers which help the individual's swallowing mechanism.

The functional exam of deglutition - FEESS^®^ - *Fiberoptic Endoscopic Examination of Swallowing Safety*, described by Langmore et al. (1988)^10^, is a change in the fiberoptic nasal-pharyngo-laryngoscopy already used for decades in ENT practice. Such exam enables us to detect aspiration and establish the safety in oral feeding by means of directly observing deglutitions through fiberoptics.

In our clinic, we chose to call it deglutition videoendoscopy (DVE)^11^. DVE is an inexpensive, practical and efficient test used to assess swallowing, and it can be done in children since the first days of life all the way to adults, and in any setting (inpatients, patients in ICU or in *homecare*). It has a good diagnostic agreement when compared to other methodologies^8^. It can be done repeated times, in a sequential manner, and it enables one to monitor patient evolution, to safely remove nasogastric tubes, and document the procedure; to contraindicate oral feeding or the very realization of other tests to assess deglutitions, as well as to help prevent aspiration pneumonia^3^, ^14^, ^15^.

The main aspects observed during a functional speech therapy evaluation are the triggering of the deglutition reflex; the feeling of stuck food and the “wet” voice/cough/ hawking. These aspects may be respectively correlated with the deglutition reflex (white out phase), presence of pharyngeal residue and penetration/aspiration - main results observed through DVE^16^, ^17^, ^18^.

We decided to standardize the food given to the patient, so as to be able to have a uniform protocol which could facilitate the evaluation process and enable us to carry out more homogeneous scientific studies^19^, ^20^, ^21^.

We numbered the steps used for an anatomical and functional assessment of the nasal cavities, the mouth, velopharyngeal closure, pharynx, larynx, by means of complementary, subjective and objective assessment, providing examiners with good information concerning structural and functional integrity of the oropharynx, as well as the sensitivity and protection reflexes - paramount for a safe deglutition^22^. We observed: mechanical obstructions, tongue movement changes, glottic closure changes, salivary stasis in the oral cavity, pharynx and larynx, overt salivary aspiration or overt involvement of the local sensitivity. We could stop the assessment at any time, without exposing the patient to the risk of aspiration of the dye food samples^10^, ^22^, ^23^, ^24^.

During the functional assessment of deglutition by DVE, it was possible to obtain further details on the pharyngeal phase of deglutition, which corresponds to the intersection phase between the airways and digestive tract, where the intactness of the protective mechanisms of the airways is paramount to prevent aspiration. It was also possible to topographically locate the site of involvement, the deglutition time when the changes were more evident and significant, the consistency which was more difficult to swallow, and facilitating maneuvers which had a positive impact on the deglutition mechanism for each case. The findings resulting from these evaluations established the safety and efficacy of deglutition, enabling its classification.

The oral phase of deglutition involves the participation of the tongue muscles, being the most responsible for pushing the food cake towards the pharynx and, consequently, the key for the efficacy of the deglutitions reflex triggering and that of the entire pharyngeal phase^25^. More frequent findings of changes to the oral phase are associated to inadequate tongue function, tongue hesitation, reduction in tongue mobility, missing teeth, misfit prosthesis and changes to lip sealing^26^. Issues associated with a reduction in oral sensitivity are considered important, because they impact the entire oral phase^27^. Literature data consider the impaired tongue push to be associated with the occurrence of early escape, changes to tongue base mobility and post-swallowing deglutition^28^. It is known that the VESS test does not assess the oral phase of swallowing in a very detailed way, and information can be obtained indirectly by observing the early escape and changes to tongue-base mobility. At this stage it is the clinical assessment that will provide information on the voluntary control associated with tongue movement, the preparation of the food cake in the oral cavity and its transportation to the pharynx, enabling an effective assessment of the oral phase of deglutition.

Deglutition's pharyngeal phase involves a complex integration of neuromuscular and sensorial modes. The most relevant indication for VESS is associated to a detailed evaluation of deglutition in this stage, which contains the main mechanisms of airway protection, confirming the occurrence of penetration or silent aspiration in individuals who do not have clinical signs or symptoms^29^, ^30^.

To study deglutition disorders we need a multidisciplinary team, and in this context we stress the joint work of otolaryngologists and speech therapists. The importance of employing the protocol is to have well established parameters to propose a classification concerning the degree of dysphagia, to help the professionals better share ideas, to assess the best treatment option for each case and to objectively assess patient evolution.

## CONCLUSIONS

Joint ENT and Speech evaluation concerning dysphagic patients under a given protocol enables a careful and complementary approach of the dysphagic patient in relation to etiology, approach definition and patient follow up. Clinical evaluation proved to be a good tool to track changes in deglutition, while the VESS enabled a more objective diagnosis, helping the examiner understand the pathophysiology and to treat the dysphagic patient.

**Attachments I**

**INSTITUTION**

**ENT / SPEECH THERAPY WARD**

**DYSPHAGIA GROUP**

**DEGLUTITION FUNCTIONAL EVALUATION PROTOCOL**

**DEGLUTITION VIDEOENDOSCOPY**

**I) IDENTIFICATION**

name:........................................................................................................ ID:............................

Age:............................. birth date:..............................................date:............................

Address:.................................................................................................. telephone: .......................

Occupation:.................................................................. Companion:..................................................

DVD........................... Track.........................

**II) ANAMNESIS**

**Diagnostic:**

**Ward of Origin:**

**Complaint:**

**History of past illnesses:**

**Prior treatment and exams (clinical surgical, chemotherapy, radiotherapy):**

**General health (neuro, cardio, gastro, pneumo, allergies, hearing):**

**Medication:**

**Treatment by other professionals:**

**Habits**

( ) Smoking, how long............................................. ( ) Alcohol drinking, how long......................................

**DEGLUTITION**

**Oral Phase Pharyngeal Phase**

( ) difficulties chewing L / P / S( ) cough ( ) dry( ) productive L / P / S

( ) food sticks to the mouth ceiling L / P / S ( ) gagging L / P / S

( ) delay to start deglutitions L / P / S( ) hawking L / P / S

( ) food escaping through the lips L / P / S( ) feeling of food stuck L / P / S

( ) pain in the oral cavity L / P / S( ) difficulty to swallow L / P / S

( ) delay in swallowing L / P / S( ) pain to swallow L / P / S

( ) sialorrhea

Consistency............................................................... Quantity....................................

Posture.............................. Utensils........................ Temperature..................................

( ) changes to the appetite

( ) changes to tasting

( ) increase in meal time usual time.................................... current...................

( ) tiredness to feed

**Others**

( ) burning / heartburn / reflux ( ) intubation time..........................................................

(( ) nauseas( ) vomits( ) food returns( ) nasal reflux

( ) tracheostomy (cannula, #, cuff).............................

( ) ) weight loss usual weight................. current weight................BMI............... height:......

( ) dry mouth( ) much saliva

( ) pneumonia how many ................................................ when......................................

**Feeding**

Per Os ( ) NGT ( ) gastrostomy/jejunostomy ( ) Mixed ( )

**III) SPEECH THERAPIST’S ASSESSMENT**

**1) General Status (motor, conscience, communication):**

**2) Oral language**

**Receptive:**

**Expressive:**

**3) Breathing (mode, type and coordination) Tracheostomy**

**4) Phono-articulatory organs**

**4.1. Morphology and Mobility**

**Table:** 

	POSTURE/ASPECT		MOBILITY		TONUS		CHANGES
Face (VII)	Normal	Changed	Normal	Changed	Normal	Changed	
Tongue (V, XII)	Papilla	Floor	Normal	Changed	Normal	Changed	
Lips (V, VII)	Closed	Open	Normal	Changed	Normal	Changed	
Cheeks (V, VI)	Normal	Dropped	Normal	Changed	Normal	Changed	
Mandible (V,VII,IX,X)	Symmetrical	Asymmetrical	Normal	Changed			
Soft Palate	Normal	Changed	Normal	Changed	Normal	Changed	
Hard Palate	Normal	Changed					

**Teeth** ( ) present( ) absent

( ) Total dental prosthesis ( ) Partial dental prosthesis

( ) Well adapted ( ) Maladapted

Current status( ) great ( ) good ( ) regular ( ) bad

**Oral sensitivity** ( ) touch( ) adequate( ) changed

( ) thermal( ) adequate( ) altered

( ) gustative ( ) adequate( ) altered

**4.2. Reflexes** Gag/vomit( ) absent( ) present

Cough( ) absent( ) present( ) efficient( ) inefficient

**4.3. Voice**

**Vocal quality - GRBASI TMF** scale utterance/a/:______

**G(grade): R (roughness): B(breathiness): A(asthenia): S(stress): I(instability):**

**Change Grade 1** mild **2** moderate **3** severe **4** extreme

**Others**

( ) normal( ) dyplophonia ( ) hypernasal ( ) wet( ) pasty( ) hyponasal

( ) bitonal( ) shaky( ) strangled ( ) whispered( ) rough

**SPEECH**

**Articulation:** ( ) precise ( ) imprecise recisa

**Speech intelligibility:**

( ) unintelligible( ) intelligible when focused ( ) partially intelligible ( ) intelligible

**Diadochokinesia rate:** PA (# pal/sec) TA (# pal/sec) KA (# pal/sec)

PA TA KA (# pal/seg)

**RECORDING (date)**:..............(spontaneous speech, prolonged utterance é, PA TA KA, phrases)

**5) Swallowing Assessment**

**5.1. Saliva**

automatic ( ) voluntary ( ) absent ( )

normal ( ) build up ( ) sialorrhea ( ) xerostomia ( )

laryngeal lift: present ( ) absent ( ) reduced ( )

gagging/cough: Y ( ) N ( )

wet voice: Y ( ) N ( )

**5.2. Food**

Body and neck posture:........................................................................................

Cuff: inflated ( ) partially inflated ( ) empty ( )

**Table:** 

CONSISTENCY	LIQUID				THICK LIQUID				PASTY				SOLID	
QUANTITY														
Utensil														
Mouth opening	nl	alt	nl	alt	nl	alt	nl	alt	nl	alt	nl	alt	nl	alt
Lip grasping	nl	alt	nl	alt	nl	alt	nl	alt	nl	alt	nl	alt	nl	alt
Tongue mobility	nl	alt	nl	alt	nl	alt	nl	alt	nl	alt	nl	alt	nl	alt
Oral transit time	nl	alt	nl	alt	nl	alt	nl	alt	nl	alt	nl	alt	nl	alt
Reflex triggering	nl	alt	nl	alt	nl	alt	nl	alt	nl	alt	nl	alt	nl	alt
Laryngeal lift	nl	alt	nl	alt	nl	alt	nl	alt	nl	alt	nl	alt	nl	alt
Gagging/cough	no	yes	no	yes	no	yes	no	yes	no	yes	no	yes	no	yes
Hawking	no	yes	no	yes	no	yes	no	yes	no	yes	no	yes	no	yes
Residue in the oral cavity	no	yes	no	yes	no	yes	no	yes	no	yes	no	yes	no	yes
Oral residue clearance	no	yes	no	yes	no	yes	no	yes	no	yes	no	yes	no	yes
Food exit through tcht	no	yes	no	yes	no	yes	no	yes	no	yes	no	yes	no	yes
Neck/lung auscult	nl	alt	nl	alt	nl	alt	nl	alt	nl	alt	nl	alt	nl	alt
Wet voice	no	yes	no	yes	no	yes	no	yes	no	yes	no	yes	no	yes
Food stuck feeling	no	yes	no	yes	no	yes	no	yes	no	yes	no	yes	no	yes
Dyspnea	no	yes	no	yes	no	yes	no	yes	no	yes	no	yes	no	yes
Nasal reflux	no	yes	no	yes	no	yes	no	yes	no	yes	no	yes	no	yes
Increased secretions	no	yes	no	yes	no	yes	no	yes	no	yes	no	yes	no	yes
Postural maneuvers	no	yes	no	yes	no	yes	no	yes	no	yes	no	yes	no	yes
AW prot. maneuvers	no	yes	no	yes	no	yes	no	yes	no	yes	no	yes	no	yes
# deglutitions														

**Legend**: Utensils: CN-straw; CP-cup; CL-spoon; S-syringe

**Observations**: (nausea/vomit and others):.....................................................................................................

Chewing:.........................Maneuvers utilized: postural:...............Airway protection:.....................

**CONCLUSION**: ( ) Normal deglutition Oral dysphagia ( ) Oropharyngeal D ( ) Pharyngeal D ( )

By consistency: ( ) Mild dysphagia ___ ( ) Moderate dysphagia ___ ( ) Severe dysphagia ____

General classification: ( ) Mild dysphagia ( ) Moderate dysphagia ( ) Severe dysphagia

( )dysarthria ( ) apraxia( ) aphasia ( )dysphonia ( ) dysarthrophonia ( ) other:.....................................

APPROACH:..............................................Examiner:.......................................................

**IV) ENT EVALUATION**

**1. Nasal Cavities**

Septum( ) centered ( ) deviated R ( ) deviated L ( ) non-obstructive irregularities

Mucosa( ) normal( ) pale( ) red ( ) edematous ( ) wet( ) atrophic

Turbinates ( ) normal ( ) hypertrophic

**2. Rhinopharynx:**

Mucosa( ) normal( ) pale( ) red( ) edematous ( ) wet( ) atrophic

Tube ostia( ) free( ) obstructed

**3. Velopharyngeal sphincter:**

Phonation( ) complete closure ( ) local incomplete closure:...............

( ) coronal( ) sagittal( ) circular ( ) circular with Passavant ring

( ) insufficient( ) incompetent

Deglutition ( ) complete closure ( ) local incomplete closure:...............

( ) coronal( ) sagittal( ) circular ( ) circular with Passavant ring

( ) insufficient( ) incompetent

**4. Hypopharynx (IX,X,XII)**

Tongue base - mobility ( ) adequate ( ) altered.............................

Valleculae( ) normal( ) lesion ( ) saliva stasis

Epiglottis( ) normal( ) omega( ) lesion.................................

Arytenoids ( ) normal( ) hyperemia( ) edema grade.................

Interarytenoid region ( ) normal( ) hyperemia( ) edema grade.................

Pyriform sinuses ( ) free( ) obstructed ( ) salivary stasis ( ) R ( ) L

Pharyngeal sensitivity ( ) normal( ) reduced ( ) absent ( ) increased

Mucosa ( ) normal( ) edematous ( ) rough ( ) pachydermia

**5. Larynx** vocal folds ( ) mobile( ) others................................

( ) paresis( ) R( ) L

( ) paralysis( ) R( ) L

( ) arching ( ) R( ) L

( ) atrophy ( ) R( ) L

( ) lesion.................................( ) R( ) L

Ventricular folds ( ) normal ( ) hyperconstriction ( ) R( ) L

Laryngeal asymmetry ( ) yes ( ) no

Laryngeal sensitivity upon a mechanical stimulus:

Epiglottis ( ) normal( ) changed

Aryepiglottic fold ( ) normal ( ) changed

arytenoids ( ) normal ( ) changed

vocal folds ( ) normal ( ) changed

ventricular bands ( ) normal ( ) changed

saliva aspiration ( ) present ( ) absent

subglottis( ) normal ( ) changed

**6. Glottic closure** ( ) complete ( ) incomplete ( )consistent ( ) inconsistent

( ) anterior spindle-like cleft ( ) total spindle-like cleft ( ) hourglass-shaped cleft

**7. VESS Table**

**Table:** 

				Reflex trigger						Residue							
Consistency	Quantity	Nasal reflux	Early escape	Normal	Delayed	Absent	Penetration	Aspiration	Cough reflex	Tongue back	Valleculae	S.P.D	S.P.E	Post. wall	# deglutitions	# clearances	Postural maneuver

**Table:** 

Liquid	1 ml	S	N	S	N	S	N	S	N	S	N
		S	N	S	N	S	N	S	N	S	N
		S	N	S	N	S	N	S	N	S	N
	3 ml	S	N	S	N	S	N	S	N	S	N
		S	N	S	N	S	N	S	N	S	N
		S	N	S	N	S	N	S	N	S	N
	5 ml	S	N	S	N	S	N	S	N	S	N
		S	N	S	N	S	N	S	N	S	N
		S	N	S	N	S	N	S	N	S	N
	10 ml	S	N	S	N	S	N	S	N	S	N
		S	N	S	N	S	N	S	N	S	N
		S	N	S	N	S	N	S	N	S	N
Liquid iquid	1 ml	S	N	S	N	S	N	S	N	S	N
		S	N	S	N	S	N	S	N	S	N
		S	N	S	N	S	N	S	N	S	N
	3 ml	S	N	S	N	S	N	S	N	S	N
		S	N	S	N	S	N	S	N	S	N
		S	N	S	N	S	N	S	N	S	N
	5 ml	S	N	S	N	S	N	S	N	S	N
		S	N	S	N	S	N	S	N	S	N
		S	N	S	N	S	N	S	N	S	N
	10 ml	S	N	S	N	S	N	S	N	S	N
		S	N	S	N	S	N	S	N	S	N
		S	N	S	N	S	N	S	N	S	N
Pasty	½ colher	S	N	S	N	S	N	S	N	S	N
		S	N	S	N	S	N	S	N	S	N
		S	N	S	N	S	N	S	N	S	N
	1 colher	S	N	S	N	S	N	S	N	S	N
		S	N	S	N	S	N	S	N	S	N
		S	N	S	N	S	N	S	N	S	N
Solid	1/4	S	N	S	N	S	N	S	N	S	N
		S	N	S	N	S	N	S	N	S	N
		S	N	S	N	S	N	S	N	S	N

**CONCLUSION:** ( ) Normal deglutition ( ) Oral dysphagia ( ) oropharyngeal dysphagia ( ) Pharyngeal dysphagia ( )

**By consistency**: ( ) Mild dysphagia ______ ( ) Moderate dysphagia _______ ( ) Severe dysphagia _______

**General classification**: ( ) Normal deglutition ( ) Mild dysphagia ( ) Moderate dysphagia ( ) Severe dysphagia

**APPROACH**: ( ) Speech therapy ( ) education ( ) reassessment ( ) High ( ) other:____________________

**Examiner**: ________________________________
